# Aligning Indigenous and Western Concepts of Health Resource Decision Making in a Western Canadian First Nations Context

**DOI:** 10.1007/s40258-025-01004-4

**Published:** 2025-09-21

**Authors:** Aidan Neill, Stephanie Montesanti, Lea Bill, Barbara S. E. Verstraeten, Rhonda C. Bell, Richard T. Oster, Arto Ohinmaa, Mike Paulden

**Affiliations:** 1 School of Public Health, University of Alberta, Edmonton, Canada; 2 Collaborative Applied Research for Equity in Health Policy and Systems (CARE) Lab, School of Public Health, University of Alberta, Edmonton, Canada; 3 Alberta First Nations Information Governance Center, Calgary, Canada; 4 Department of Agricultural, Food and Nutritional Sciences, University of Alberta, Edmonton, Canada; 5 Indigenous Wellness Core, Alberta Health Services, Edmonton, Canada

## Abstract

**Background:**

Western health economic evaluation tools often fail to reflect the relational, collective, and holistic perspectives that underpin Indigenous concepts of health. These limitations pose challenges when applying Western measures in Indigenous contexts. The individualistic foundation of the Western definition of health and the values that inform it are significantly different from the community-based values typically found in Canadian Indigenous communities. For health economics to effectively support Indigenous health decision making, a values-based approach should initially be undertaken to identify conceptual commonalities with Western perspectives.

**Aims:**

This study aimed to develop a conceptual framework that identifies shared elements between Western and First Nations understandings of health resource decision making, with the goal of supporting the creation of culturally meaningful health outcome measures.

**Methods:**

Through a Health Economics Technical Advisory Group (HE-TAG) in Alberta, Canada, co-led by Indigenous and non-Indigenous researchers, we conducted a conceptual exploration of health resource decision making. Fourteen HE-TAG sessions held between July 2021 and June 2023 were transcribed and analyzed using a hybrid approach—combining Q methodology, thematic analysis (Braun & Clarke), and Walker and Avant’s concept analysis.

**Data:**

Transcripts from 14 HE-TAG sessions provide the qualitative data upon which analysis was conducted. Sessions were held online using virtual meeting technology, and recordings were transcribed and analyzed.

**Results:**

Indigenous and Western conceptual frameworks allow for a common understanding of health resourcing. Indigenous community and culture and Western economic evaluation and social determinants of health were the four main observed themes, each of which contained two subthemes which differentiated between concepts of ‘health.’ Five concepts were found to resonate between Indigenous and Western themes when exploring health resource thinking: *values*, *holism*, *time*, *resources*, and *context*. Concepts and themes were mapped to illustrate common approaches to understanding health resource decision making.

**Conclusions:**

This Indigenous-informed research aligns concepts of resource decision making by showing the thematic backgrounds of First Nations and Western thinking, which are linked by the common concepts of *values*, *holism*, *time*, *resources*, and *context*. Centering future community engagement on these shared concepts while grounding them in community-generated health value sets can advance the development of novel, culturally relevant health outcome measures.

## Key Points for Decision Makers

**Table:** 

Key findings and implications of this research include:
Generating a thematic basis for resource decision making in a Western Canadian First Nations context.
Producing a values-based framework for future development of culturally relevant health outcome measures with Canadian First Nations communities.
Demonstrating thematic connections between Indigenous and Western ways of understanding resource decision making using the concept of health.
Practical applications of these findings could involve using the framework during community co-development of health outcome measures, demonstrating the centrality of holistic values to health technology assessment agencies when evaluating outcomes with Indigenous communities, and the use of First Nations health values in priority setting and other economic exercises in community. While the framework is context specific, this approach to generating culturally meaningful thematic representations of health could potentially be more widely applied.

## Introduction

Efforts to improve health outcomes for Indigenous Peoples in Canada, such as those outlined in Call to Action #19 of the Canadian Truth and Reconciliation Commission [4], require meaningful engagement with Indigenous perspectives and knowledge systems. Disparities in infant mortality, maternal health, chronic disease, and access to health services persist, underscoring the need for approaches that are not only equity-oriented but also culturally grounded. However, the tools used to measure and evaluate health interventions—particularly within the field of health economics—often fail to reflect Indigenous worldviews. Western preference-based measures of health have been found unsatisfactory in their application to Indigenous populations [5, 6], and this is mainly due to the individualistic versus community value differences between the two worldviews [7]. To effectively measure Indigenous health improvements within a culturally appropriate framework, it is essential to conceptually map out the similarities and differences in health decision making across the Indigenous and Western perspectives. A shared foundation for future community involvement was established through a conceptual exploration of Western health economics alongside First Nations perspectives on health resource decision making within a Canadian province.

Effort has been made throughout this manuscript to use terminology that is both respectful and appropriate to the context of the research. The terms *Indigenous*, *Aboriginal, First Nations*, *Inuit*, *Indian*, and *Métis* are used with care and intent. *Indigenous* has been applied in circumstances where *First Nations*, *Inuit*, and *Métis* groups are collectively implied. This term has also been used in its most applied international context such as that used by UNDRIP [8]. In this paper, we use the term *First Nations* intentionally, reflecting the specific context and partnerships of this research, which is conducted in collaboration with First Nations communities participating in the Canadian Institutes of Health Research (CIHR) Indigenous Healthy Life Trajectories Initiative (I-HeLTI) study. As such, the health value sets referenced throughout this work are identified as *First Nations*. Their connection to Indigenous knowledge systems is also recognized and affirmed [9]. The specific use of language is intended to clarify the approaches taken, while suggesting potential generalizable applications. While it is hoped that wider applications of this work may be undertaken in future, there is no assumption that a pan-Indigenous approach will be taken with the specific results, which are based on a Western Canadian First Nations context.

### Background

#### Western Health Economics and Its Limitations in Indigenous Contexts

Although the Royal Commission on Aboriginal Peoples (RCAP) suggested a potential convergence between Indigenous health concepts and contemporary research on the social determinants of health (SDH) [10], there remain unexplored differences between Indigenous and Western health paradigms. In health economics, *health value sets* are foundational tools used to quantify the relative importance of different health states. Derived from general population preferences, these value sets assign weights to health states—typically assessed through surveys using methods such as time trade-off or discrete choice experiments—and are used to generate summary measures like quality-adjusted life years (QALYs) [11]. These measures inform cost-effectiveness analysis and guide resource allocation and policy decisions.

However, standard health value sets have limited cultural validity when applied in Indigenous contexts. They often omit domains that are central to Indigenous understandings of health, such as cultural identity, connection to land, intergenerational relationships, and spiritual wellness. As a result, they may inadequately reflect Indigenous health priorities and values.

To enable more culturally meaningful evaluation of health interventions with Indigenous communities, a different approach is needed—one that begins by identifying areas of conceptual commonality between Indigenous and Western perspectives. This paper explores such commonalities to inform the co-development of culturally relevant health value sets grounded in Indigenous worldviews.

Indigenous scholars are concerned that standard forms of health state measurement fail to capture the breadth of Indigenous experiences [12]. Non-Indigenous researchers alike have voiced concerns over the lack of relevant domains used for economic evaluation of health programming, that are connected to the social and community aspects of health [5]. This raises fundamental questions about the validity of these measures in reflecting what constitutes Indigenous health [6].

**Fig. 1:**
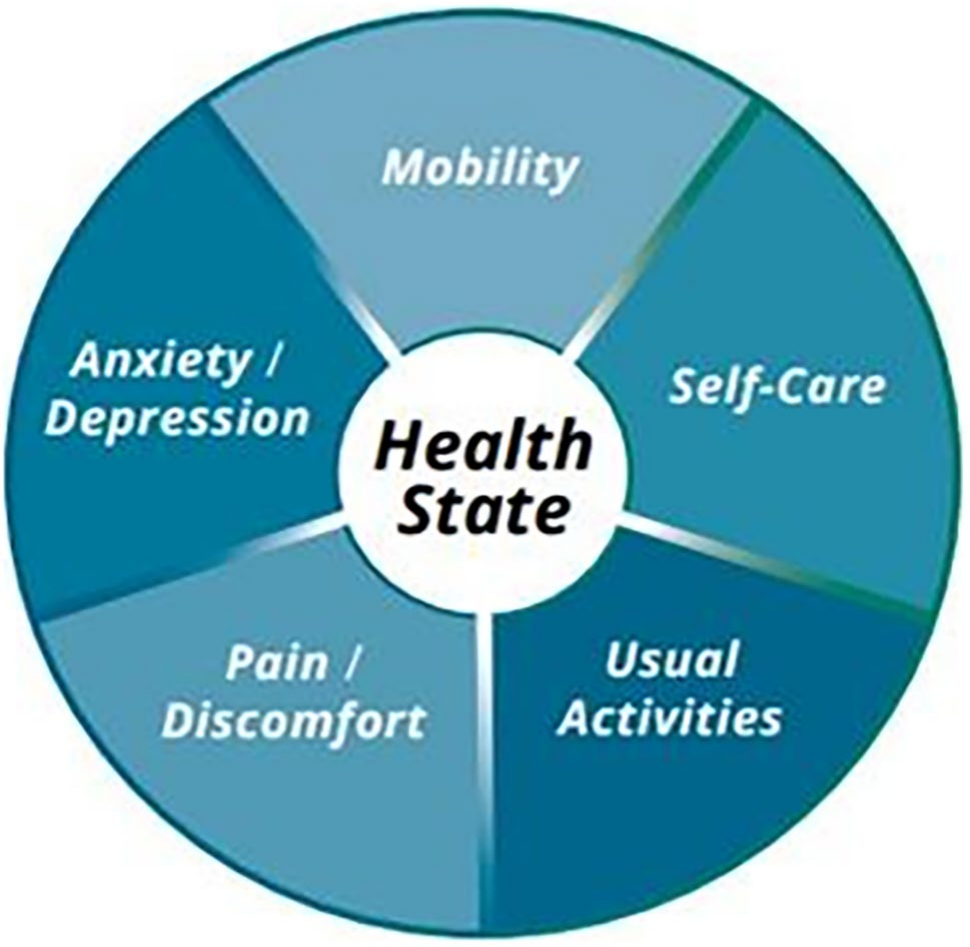
The five domains of the EQ-5D

Several preference-based measures (PBMs), such as SF-6D, SF-12, SF-36, AQoL-4D, in addition to the EQ-5D, have been used with Indigenous populations; however, these have been found not to effectively incorporate Indigenous concepts of quality of life (QoL) [13]. The EQ-5D utilizes a five-domain descriptive classification of health that includes mobility, self-care, usual activities, pain/discomfort, and anxiety/depression [14] (Fig. 1). One Indigenous-specific HRQoL tool was identified from Canada, which used surveys and Likert scales, but it was not preference-based [5]. In Australia, the ‘Good Spirit, Good Life’ tool and framework identify relevant Indigenous QoL domains, but it is likewise not developed into a preference-based measure [15]. Limited evidence was found of the suitability of applying current PBMs with Indigenous populations [6]. While evidence and suitability of PBMs with Indigenous populations is limited, priority setting can be used to address the differing concepts of health between Indigenous and non-Indigenous groups [7]. Criteria for priority-setting exercises are based upon a discrete set of values from which decisions can be made [16]. However, the implementation of priority setting with Indigenous groups from a health economic evaluation vantage has not been widely undertaken [17].

#### First Nations Health Value Systems

**Fig. 2:**
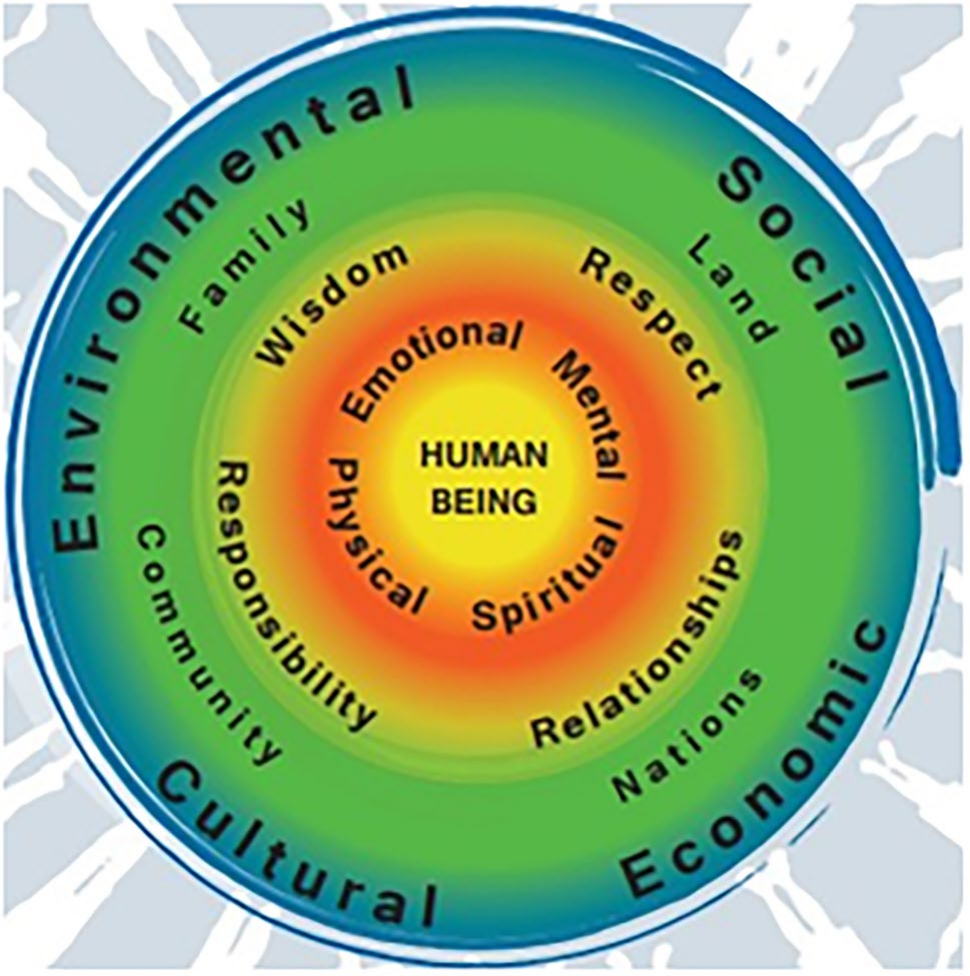
First Nations Health Authority (FNHA) perspective on health and wellness

Two prominent First Nations frameworks in Canada offer culturally grounded understandings of health that differ significantly from Western paradigms. The First Nations Health Authority (FNHA) in British Columbia developed the *‘First Nations Perspective on Health and Wellness’* [18], a holistic model represented by concentric circles. In this health value set (Fig. 2), the individual is at the centre, surrounded by layers that reflect physical, emotional, spiritual, and mental wellness; followed by principles such as respect, wisdom, and responsibility; and expanding outward to include family, community, land, and the broader environment. The outermost circle shows a connected community holding hands—symbolizing intergenerational ties and collective well-being. This model emphasizes relationality, interconnectedness, and balance as core to health.

**Fig. 3:**
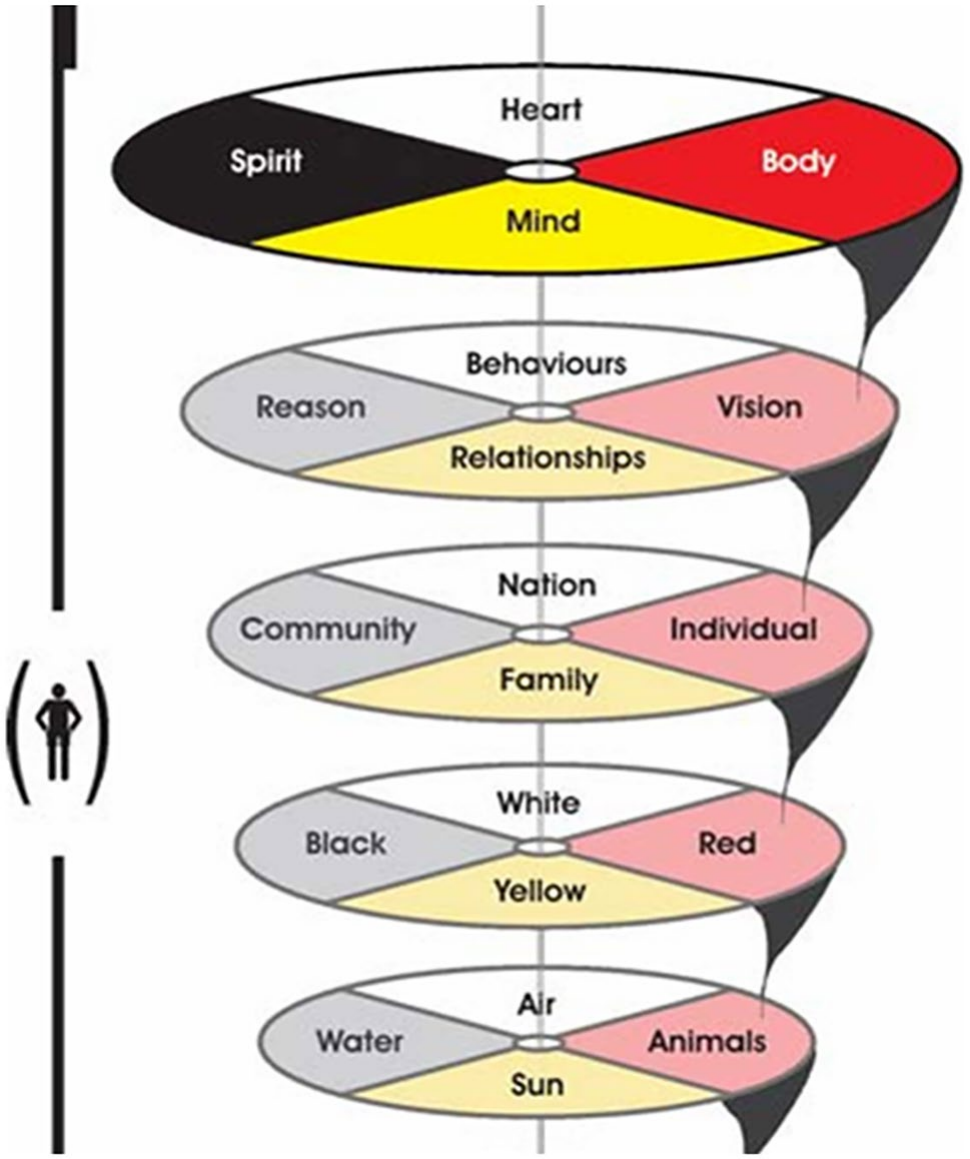
First Nations Information and Governance Center (FNIGC) cultural framework

Another well-articulated health value set is that of the First Nations Information and Governance Center (FNIGC)—*“Total Health of the Total Individual within the Total Environment”* (Fig. 3)—which is also referred to as the Regional Health Survey Cultural Framework of Total Health [9]. This Cultural Framework has three distinct components: (1) the total health of the total person set within a 4-direction model, containing body, spirit, heart, and mind; (2) the total environment seen within a 4-direction model, containing individual, culture, community, and family; and (3) the ‘ingredients’ for a healthy Indigenous community which include relationship, harmony, ways of living, sovereignty, culture, and environment. As we observe in both the FNHA and the FNIGC health value sets, they are complex and multi-layered, demonstrating multiple connections among elements, and view the health of the individual as intricately connected to the health of the community and the broader environment. FNIGC’s four-direction model is also represented by a multi-dimensional medicine wheel. Indigenous values of health are fundamentally and inextricably bound to the physical and social environment which surrounds and connects the individual. Though these are not ‘value sets’ in the economic sense, they represent guiding principles and priorities for health that can inform the development of culturally relevant outcome measures for economic evaluation.

#### Social Determinants of Health

Indigenous health value systems closely align with broader social determinants of health (SDH), including housing, education, income, access to traditional lands, and historical trauma. These determinants, shaped by colonial policy and systemic inequities, continue to affect the health and well-being of Indigenous Peoples [19]. Furthermore, closely related to SDH is the concept of a social gradient, that is, population inequalities resulting from social status disparities. Research tracing the social gradient to primary and secondary determinants of health suggests a path model from SDH to the measurement of health outcomes [20]. The pathway from primary SDH (socio-demographic factors) through secondary SDH (control, self-esteem, social support & involvement), which can be assessed, demonstrates the complexity of SDH and the ability to measure outcomes with health and wellness indicators. As the SDH show a broader aspect of health, this suggests the need for intersectoral approaches that transcend the boundaries of the health care system [21].

#### Holism and Health Evaluation

Limited applications have been made within health economics to adopt a more holistic approach to health outcomes [22, 23]. Recently, the adoption of holistic approaches has been recommended when addressing SDH rooted in structural, systemic factors based on the inequity of power [24] experienced by Indigenous peoples. Holism is a defining feature of Indigenous worldviews, viewing health as more than the sum of its parts [25]. It recognizes the interconnectedness of physical, emotional, spiritual, and mental dimensions, as well as the interdependence between individuals, families, communities, and the land [26]. Holistic thinking recognizes the complex interconnectedness and relatedness of elements, avoiding the summary reduction of these elements into analytic, abstract representations, thereby seeking a middle ground between opposing forces [27]. Though rarely integrated into Western economic evaluation, holistic models are gaining traction, particularly in Indigenous health research. For example, Australia’s “Fabric of Aboriginal and Torres Strait Islander Wellbeing conceptual model” [28] and the “What Matters 2 Adults” (WM2A) initiative aim to develop wellbeing measures that reflect Indigenous conceptions of quality of life [29, 30]. These efforts illustrate growing recognition of the need to embed holistic values into measurement and decision making.

#### Systems and Complexity Theory

Health systems that serve Indigenous communities are embedded within broader social, political, and historical systems [31, 32]. As such, systems thinking and complexity theory offer valuable tools for understanding the dynamic interactions between policies, institutions, community conditions, and health outcomes. Complexity-informed approaches are particularly well suited to Indigenous health contexts. They highlight dynamic complexity, interrelatedness of system elements, feedback loops, boundary critique, adaptivity, and non-linear causality—aligning with Indigenous notions of interdependence and relational accountability [33]. These approaches also offer ways to consider counterintuitive methods, use recursive techniques, understand conceptual limits, and identify when specific policy actions or methods are ineffective. In relation to Indigenous health, complexity theory challenges the notion of top-down control, exposes multiple perspectives, sees a plurality of voices and examines relationships [34, 35]. Such approaches can help illuminate how colonial policies, funding structures, and institutional practices perpetuate health inequities. Additionally, they can inform Indigenous research [36]. Systems thinking also involves the consideration of intersectoral approaches [37].

#### Expanding the Perspective in Health Economic Evaluation

The choice of perspective in health economics paves the way for considering multi-sectoral approaches that address SDH, acknowledge systems and complexity approaches, and respond to calls for holistic methods. Adopting a broader perspective during economic evaluation allows for the inclusion of sectors beyond health care [38], and the evidence gathered through this approach could align with holistic methods that do not rely on reductive, analytic techniques. These alternative methods encompass health profiles [14], cost consequence analysis [39], social accounting matrices [40], and impact inventories [41]. Each method provides a transparent, disaggregated display of health costs and outcomes for decision makers to consider in their deliberative processes. Rather than aggregating preferences into a social function, which can pose challenges [42–44], deliberation utilizing these disaggregated methods is often preferred for enhancing decision-making dialogue [45] as it promotes consent and is perceived as holistic, open, inclusive, and uncoerced [46]. By comparison, the cost-benefit analysis (CBA), which would be less suitable for use in a holistic context, can be reductive and monetizes costs and benefits [47], which exhibits quantification bias. This may overlook values when considering outcomes. Likewise, the return on investment (ROI) method is a reductive financial metric [48], which does not consider factors beyond monetary returns.

## Methods

### Study Context: The Indigenous Healthy Life Trajectories Initiative (I-HeLTI)

This study was embedded within the Canadian Institutes of Health Research-funded, *Indigenous Healthy Life Trajectories Initiative (I-HeLTI)*, which applies a developmental origins of health and disease (DOHaD) lens to mitigating the risk of non-communicable diseases (NCDs) [49]. I-HeLTI research “utilizes traditional Indigenous knowledge to inform and reclaim positive pathways for promoting and sustaining healthy child development and measuring the well-being of children and families over time” [1]. During the second phase of I-HeLTI, a Health Economics Technical Advisory Group (HE-TAG) was established between July 2021 and June 2023 to explore the question, “Do commonalities exist between Indigenous and Western approaches to health resource decision making?” The HE-TAG’s orientation to this question stemmed from its ongoing, critical position of exploring whether health economics could practically contribute to Indigenous decision making. The focus was therefore kept on health resources during discussion. The response to this inquiry aims to inform future community partner engagements for developing a culturally appropriate, values-based measure of Indigenous health outcomes for I-HeLTI programming in the subsequent phase three, which is a 6-year cohort intervention study of community-based programs, that emphasize the roles of family and kinship central to pregnancy and childbirth. The 23 communities in British Columbia and Alberta participating in I-HeLTI include the 14 Nuu-chah-nulth (NCN) Nations, represented by the NCN Tribal Council (NTC), the four Cree Nations of Maskwacîs (CNM), and the five Cree and Dene Nations of the Regional Municipality of Wood Buffalo (CDNWB).

### Community-Based Participatory Approach

The study followed a community-based participatory research (CBPR) approach [50], grounded in relational accountability and guided by Indigenous leadership. The Alberta Co-Principal Investigators (SM, RO, RB, LB) worked closely with community partners throughout the study to ensure that the research question, design, and interpretation were informed by local perspectives and Indigenous knowledge systems.

Community input was reflected in the formation of the HE-TAG, which brought together Indigenous and non-Indigenous researchers, a Knowledge Keeper, economists, and policy practitioners to engage in dialogue around the conceptual foundations of health and evaluation. Knowledge and insights from community engagement—including advisory committees, Elders, and local health leaders—were shared with the HE-TAG to inform deliberations and ensure cultural relevance.

During the authorship of this paper, co-authors all participated in the co-production of the work. A.N. contributed health economic technical knowledge during discussions, conceived the analytical methodology, led the data analysis, and produced the framework. S.M. contributed significant conceptual framing of the approach and the results, in addition to conducting in-depth editorial improvements to the work and mentorship on qualitative and Indigenous research methodology. She also leads the HE-TAG. L.B. shared her perspective as a Knowledge Keeper and speaker of the Cree language during the many HE-TAG sessions, which she expertly guided around topics of Indigenous knowledge. B.V. was integral to the production of HE-TAG data by producing the session transcripts on which the analysis was done; B.V. also provided extensive editorial improvements to the work. R.B. attended all HE-TAG sessions and provided strong continuity to the discussions while also providing many critical edits to the final work. R.O. provided tremendous encouragement and support during the final phases of the writing, and the two-eyed seeing approach can be credited to his suggestion. M.P. was the expert health economist who anchored the Western conceptual perspective for health resource decision making.

### HE-TAG Process and Data Generation

The HE-TAG held 14 virtual sessions between July 2021 and June 2023. Each session was approximately 1 hour and was conducted via Zoom^™^. The key question, “do commonalities exist between Indigenous and Western approaches to health resource decision making?” was explored through discussion with the members of the HE-TAG. Discussions focused on identifying resonances and tensions between Indigenous and Western health concepts, particularly in the context of health resource allocation and outcome measurement. The discussions occurred over the course of 2 years and yielded a set of meeting transcripts, which were subsequently analyzed. Sessions were recorded with the seven HE-TAG participants’ consent and transcribed verbatim for analysis. A.N., S.M., L.B., B.V., R.B., R.O., and M.P. were the members of the HE-TAG. Recurrent themes were observed throughout the discussion records by observing topic and word frequency. Themes were then further analyzed and defined into sub-themes. The transcripts were then re-analyzed to generate a set of concepts related to the initial health concept. This process was done recursively until a framework was produced that reflected the overall meaning of the HE-TAG discussions.

### Ethical Considerations

Ethics approval for the I-HeLTI project is covered under Alberta Research Information Services (ARISE) ethics protocol number 97455, led by Principal Investigator Dr Stephanie Montesanti. The study is titled *Restoring Healthy Family Systems in Indigenous Communities*. This research was conducted in accordance with the Declaration of Helsinki [2] and Chapter 9 of the Tri-Council [3].

### Qualitative Approach

A multi-method qualitative approach was applied to analyze the I-HeLTI HE-TAG data, combining discourse analysis, thematic analysis, and concept analysis to capture both the surface structure and deeper conceptual content of the discussions. This layered approach enabled the exploration of how participants articulated, understood, and negotiated Indigenous and Western perspectives on health resource decision making. The analytical process began with a structural-semantic discourse analysis [51–53], which examined how meaning was constructed through language, tone, and patterns of speech. This bottom-up approach helped identify recurring patterns and connections between ideas. Signifiers of meaning were found in key words that formed patterns, pathways, and structures upon analysis. This phase was informed by principles from ‘Q methodology,’ which focuses on capturing regularities in meaning making based on semantic elements during initial transcript exploration [52]. After the initial discourse analysis, a thematic analysis followed Braun and Clarke’s six-step process [54] to (i) review transcript results; (ii) generate initial codes; (iii) identify recurrent themes; (iv) review themes; (v) define and name themes; and (vi) codify and report results. Thematic insights were then integrated into a concept analysis following step four of Walker & Avant’s model (defining attributes) [55] (Table 1), which is also the “heart of concept analysis” [56]. Together, these methods provided a robust and recursive analytic strategy. This triangulated approach supported the development of a conceptual framework that can inform the design of culturally resonant health outcome measures.

**Table 1:** Application of Walker and Avant’s concept analysis

Step	Walker and Avant’s concept analysis	I-HeLTI HE-TAG*
1	Select a concept	‘Health’
2	Determine purpose of the analysis	Identify conceptual or thematic overlap
3	Identify all uses of the concept	Within I-HeLTI session transcripts
4	Determine the defining attributes	Using Braun and Clarke’s system
5	Identify the model case	I-HeLTI HE-TAG discussions
6	Identify other cases	Not required—limited to single case HE-TAG
7	Identify consequences	Analyze conceptual findings from discourse
8	Define empirical referents	Determine any applications of analysis

* Indigenous Healthy Life Trajectories Initiative Health Economics Technical Advisory Group

**Fig. 4:**
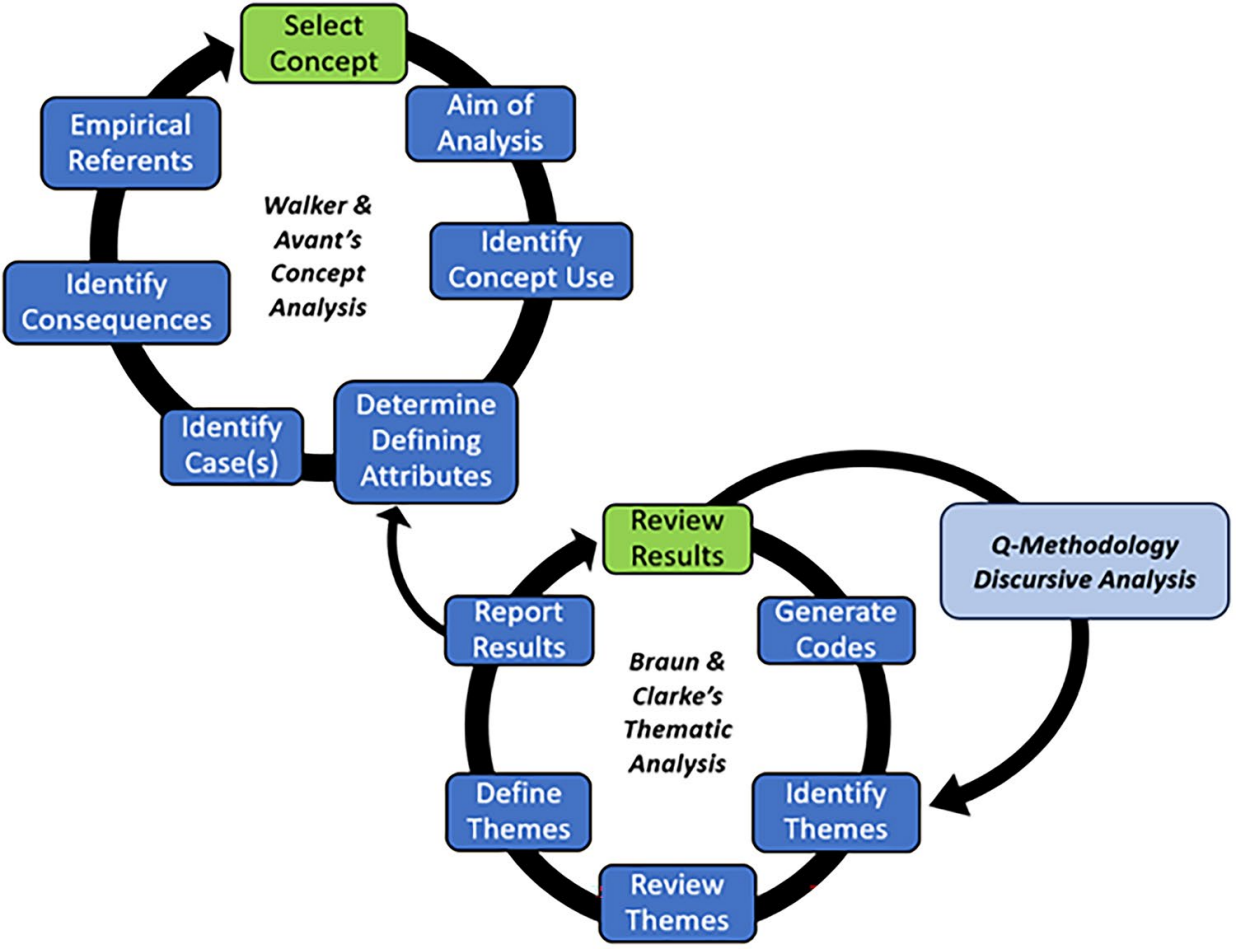
Hybrid analytical model for thematic and concept analysis

Discursive, thematic, and concept analysis proceeded circularly and recursively to produce the results (Fig. 4).

### Data Analysis

Data analysis was conducted on 14 1-hour session discussions of the HE-TAG over a 2-year period (2021–2023). The six members of the HE-TAG participated in the online sessions, which were recorded using digital teleconferencing technology (Zoom^™^) and subsequently transcribed (BV) for review and analysis. Transcripts were analyzed, and data was charted using Microsoft Excel. Codes, themes, and conceptual patterns were refined through repeated review and collaborative interpretation among co-authors. The analysis followed a recursive, multi-step process aligned with the integrated methodology described in Section 2.5. Discourse analysis identified key signifiers and discursive structures; thematic analysis mapped recurrent ideas and meanings; and concept analysis distilled defining attributes of ‘health’ across both Indigenous and Western paradigms. The lead author (AN) returned to the transcripts multiple times to refine themes, validate interpretations, and ensure the integrity of meaning, particularly in relation to Indigenous knowledge and values. A.N. and S.M. met regularly to review and identify themes. Analytical decisions were reached through consensus and relational dialogue with the HE-TAG members, reflecting a commitment to Indigenous-informed research practices.

## Results

### Conceptual and Thematic Framework

The analysis of HE-TAG session transcripts revealed a conceptual and thematic framework that maps areas of alignment and divergence between Indigenous and Western understandings of health resource decision making. Table 2 presents the concepts of Indigenous and Western ‘health’ and their respective thematic counterparts, based on the HE-TAG sessions transcript data and analysis. There are two main themes under Indigenous health (*community* and *culture*). The *community* theme has the following four subthemes: (1) ‘strengths’, (2) ‘leadership’, (3) ‘funding impacts’, and (4) ‘policy impacts.’ The *culture* theme contains four subthemes, based on Indigenous (1) ‘knowledge’, (2) ‘language’, (3) ‘medicines’, and (4) ‘values.’ The Western ‘health’ concept contains two themes (*economic evaluation* and *social determinants of health*). The *economic evaluation* theme contains the following four subthemes: (1) ‘measurement’, (2) ‘costs and resourcing’, (3) ‘outcome measures’, and (4) ‘evaluation methods.’ The *social determinants of health* (*SDH*) theme has four subthemes: (1) ‘engagement’, (2) ‘investment’, (3) ‘equity’, and (4) ‘intersectorality.’

**Table 2:**
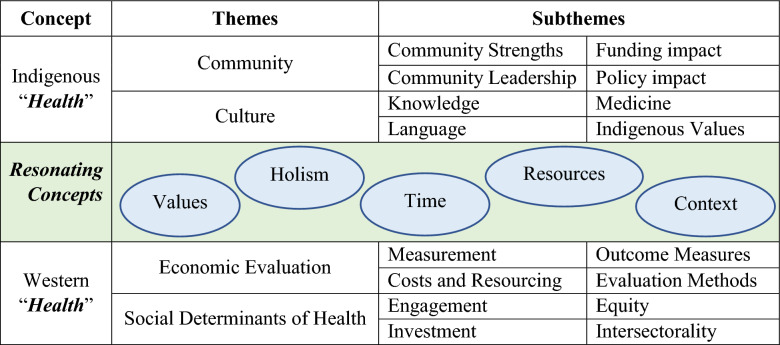
Health resourcing concept and thematic framework

Table 2 also presents five common concepts shared between health resource decision making from Indigenous and Western perspectives. These are *values*, *holism*, *time*, *resources*, and *context*. In both knowledge systems, ‘health’ possesses additional conceptual characteristics, including centrality of values, holism, consideration of time, resource consumption, and context specificity. The following thematic summaries represent consensus commentary from HE-TAG participants during the 14 sessions which produced the discursive data.

### Indigenous Framework Themes

#### Community

The *community* theme is linked to the concept of Indigenous ‘health,’ which can be understood as having embedded ‘strengths’ and ‘leadership.’ ‘Strengths’ derive from a community of individuals with shared values and involve using strengths-based measurements of success rather than gap-based Western research approaches. The presence of community rights-holders defines strengths. Resource inputs from within the community were also understood to support values-based outcomes. ‘Leadership’ is seen as a guiding force to ensure health programming is led by community Elders who guide scientific efforts. Leadership is also provided by community advisory committees (CACs), which provide broad, culturally appropriate health resources to community members. Empowerment and sustainability of Indigenous knowledge systems are also a core element of community leadership.

The *community* theme also contained two subthemes, which reflect the impact upon Indigenous communities by Western powers. ‘Policy impact’ and ‘funding impact’ upon communities were central subthemes. Funding was seen as fragmented and uncoordinated by the Canadian federal funder, which tends to disburse program funding into many separate silos with differing reporting requirements. This decision making is external to the community and is perceived as restrictive, negatively impacting community decision making. ‘Policy impact’ is wide-ranging and originates from removal from the land, which results in inaccessibility to traditional diet and medicines. Housing is inadequate and restrictive based on federal policies. Monetary policy, which historically imposed currency upon communities, was also noted. Historical policies such as Indian Residential Schools, the Sixties Scoop (where Indigenous children were put into foster care with non-Indigenous families), and other child and family policies resulted in child removal. In general, externally mandated programming is seen to be unsuitable for communities within Indigenous knowledge systems. Both detrimental colonial funding and policy impacts from external government power sources are felt acutely by the community. The *community* theme thus has elements that are embedded within it (strengths and leadership) and elements imposed upon it from external forces (funding and policy).

#### Culture

The theme of *culture* is understood as a central determinant of Indigenous health. Subthemes include ‘knowledge’ (health is guided by intergenerational knowledge held by Elders and cultural leaders), ‘language’ (using original languages [e.g., Cree, Dene, Michif] in programming was described as crucial to healing, despite limited time and resources), ‘medicines’ (traditional medicine bundles—containing plant, mineral, and spiritual elements—were discussed as culturally rooted care tools), and ‘Indigenous values’ (wellness as not based on individual preference but shaped through collective dialogue, spiritual balance, and multi-generational relationships). Regarding ‘knowledge’, the HE-TAG learned that community advisory committees aim to integrate medicine wheel teachings into the I-HeLTI programming. This knowledge is held by community Elders, including grandparents, aunties, and uncles, who are custodians of culture and ceremony, and decisions are based on this knowledge. ‘Language’ was described as essential to delivering I-HeLTI programming when conducted in the original languages of Cree, Dene, and Michif in Alberta, as well as Coast Salish languages in British Columbia. The HE-TAG acknowledges that maintaining traditional languages can be time consuming and resource intensive for community members seeking to sustain or enhance their spoken proficiency. ‘Medicines’ and ‘Indigenous values’ are two subthemes within the *culture* theme. Medicines have a spiritual component, and medicine bundles carried by Knowledge Keepers and Elders contain mineral and plant-based medicines. Indigenous values are broadly seen as holistic and consider many aspects of health. FNHA (Fig. 2) and FNIGC (Fig. 3) traditional health value systems were discussed, highlighting that the interaction of collective/social versus individual values influences health decision making. Traditional health decisions are not based on personal preferences but often involve dialogue over time and kinship ties that connect multiple generations. There is also a growing emphasis on culturally strengthening health programs rooted in Indigenous values.

### Western Framework Themes

#### Economic Evaluation

The ‘measurement’ subtheme within the *economic evaluation* theme illustrates how the dominant language of interventions is applied when discussing the assessment of health impacts. Outcomes consider the efficacy and efficiency of an intervention. Additional elements of the ‘measurement’ subtheme include interactions among system agents, comparative control groups, and time horizons for an intervention, which may range from years to the duration of a single patient or cohort group.

The ‘costs and resourcing’ subtheme encompasses program resource inputs, resource constraints, and the definition of costs concerning time (i.e., with discounting). Costs were also primarily considered within a strict healthcare setting and generally did not involve consideration of wider costs beyond the healthcare sector.

The discussion of ‘outcome measures’ highlighted how they are predominantly reflected in summary health measures, such as the QALY, which summarizes mortality and morbidity based on general population preferences. Health ‘outcome measures’ as exemplified by the EQ-5D (Fig. 1) from a Western conceptualization are typically used for gap-based analysis with Indigenous populations, and examples of this include infant mortality rates, life expectancy, injury and illness rates, etc. Outcome measures within the Western view can consider equity or fairness in the distribution of costs, health consequences, health service availability/usage, system navigation, and care integration. In general, these measures are characterized within the Western biomedical model.

The final subtheme is ‘evaluation methods.’ During the sessions, several forms of health outcome measurement were considered, including cost-effectiveness analysis (CEA), cost-utility analysis (CUA), cost-benefit analysis (CBA), and cost-consequence analysis (CCA) (Table 3).

**Table 3:** Types of health economic evaluations

Types of evaluations	Acronym	Description of health economic evaluation type
Cost-effectiveness analysis	CEA	Outcomes expressed in natural units (e.g., life-years gained, lives saved, or clinical event avoided or achieved)
Cost-utility analysis	CUA	Outcomes expressed as quality-adjusted life years (QALYs)
Cost-benefit analysis	CBA	Outcomes expressed in monetary terms
Cost-consequence analysis	CCA	Costs and outcomes presented in disaggregated form

Adapted from CADTH, 2017

The discussion centered on how to best frame a culturally appropriate economic evaluation of the Phase Three intervention birth cohort. The inter-comparability of QALYs makes CUA a dominant method for health economic evaluation. However, unlike a CUA, the CCA allows for a disaggregated display of costs and consequences (resource inputs and health outcomes), which is more transparent for decision makers. The session transcripts identified disaggregated methods, including the CCA, as potentially more applicable to an Indigenous, holistic context.

#### Social Determinants of Health

The second of two themes under Western ‘health’ is *social determinants of health*, and it contains the four subthemes of ‘engagement,’ ‘investment,’ ‘equity,’ and ‘intersectorality.’ Starting with the ‘engagement’ subtheme, it is understood that more fundamental work needs to be done to contribute to an informed, participatory discussion in communities. It was also remarked during the HE-TAG sessions that Indigenous concepts of health and contemporary research in SDH require community engagement to make the progress that RCAP envisioned in *Volume 3, Gathering Strength* [10].

‘Investment’ is the second subtheme under the *SDH* theme. As social determinants are those conditions into which one is born, raised, and lives that produce the various health outcomes and conditions, investment is a strong driver of SDH. Examples of this could be infrastructure funding such as a water treatment plant, clean water distribution systems, housing, recreation centers, roads and grounds, and other contributors to health. ‘Investment’ in programming is a key means of addressing SDH, and the I-HeLTI programming that supports community birth workers, neonates, young mothers, and their families would be one example of a health programming investment that can positively influence SDH for multiple generations simultaneously.

‘Equity’ is another subtheme found within the *SDH* theme. Equal access to necessary resources and fairness in the distribution of health programs is central to understanding equity. ‘Investment’ in communities affects equity, and the fairness of resource allocation to communities directly influences SDH by either improving or worsening access and fairness. Outcome-based health program funding can help narrow the equity gap. Addressing the greatest needs and producing the greatest benefits was central to evidence from session data. Lastly, policy structures can either improve or worsen population equity.

The final subtheme of ‘intersectorality’ involves the overlap of health programming with other programming areas, such as education and housing. There are also health impacts of a community’s interface with the justice system and child and family services when children are apprehended and removed from their home community. The components of non-health sectors have a direct influence on community capacities, which also contribute to health outcomes.

### Resonating Concepts Across Knowledge Systems

**Fig. 5:**
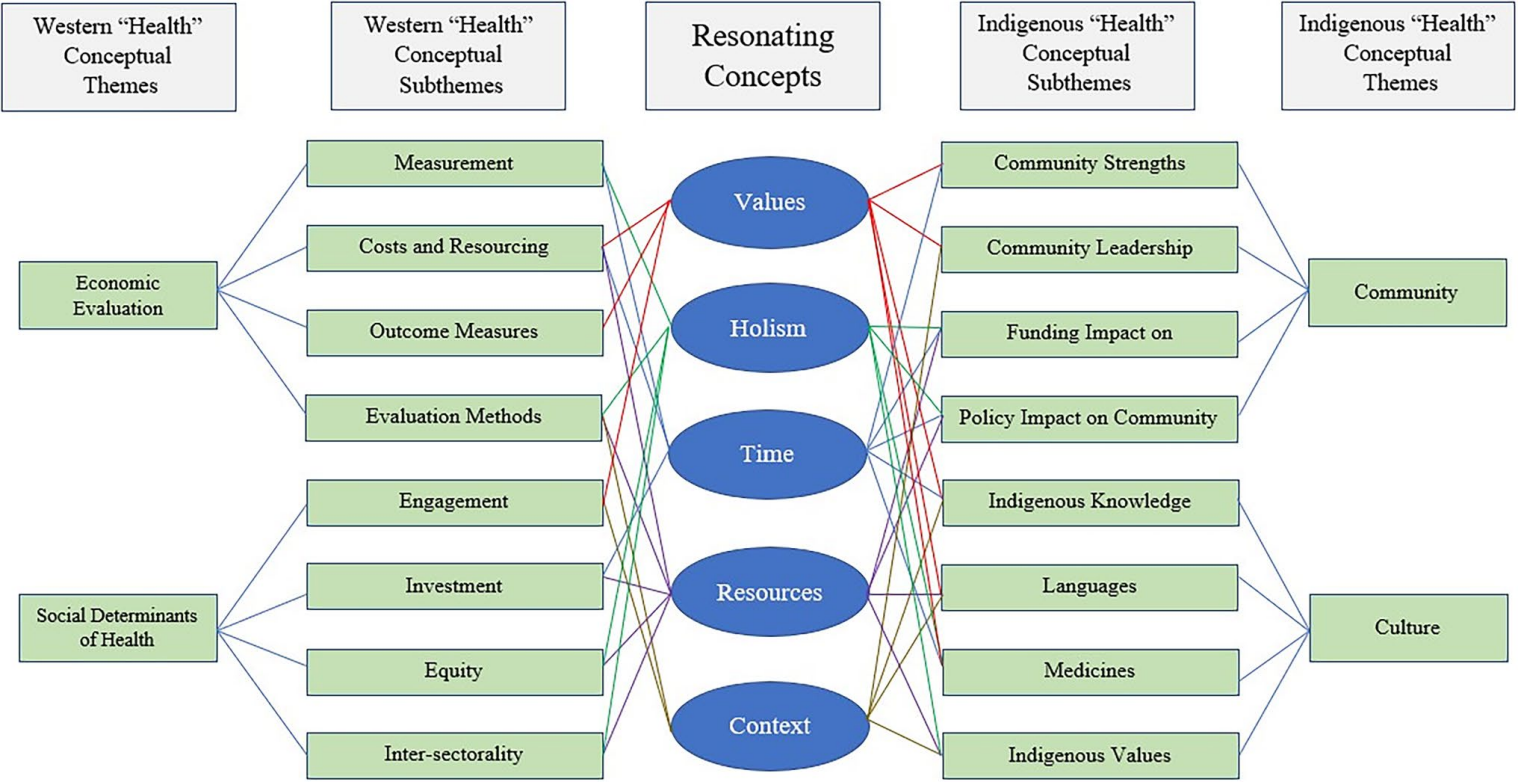
Framework of five resonating concepts between Indigenous and Western thematic health knowledge systems

Through recursive analysis, five core concepts resonated across both Indigenous and Western knowledge systems: *values*, *holism*, *time*, *resources*, and *context* (Table 2 and Fig. 5). *Values* underpin decision making in both systems, though the basis and expression of those values differ. Values are central to both Western and Indigenous systems. Health is understood as *holistic* in both frameworks, though Western evaluation tends to disaggregate while Indigenous approaches emphasize interdependence. Interdependence is a foundational principle that underscores how well-being is co-created and sustained through reciprocal responsibilities and connections. In this view, a person’s health cannot be understood apart from their relationships with others, with the land, and with the spiritual realm. Western models rely on time discounting and linear temporality; Indigenous worldviews often adopt cyclical or generational understandings of *time*. Both perspectives acknowledge the need to allocate *resources* efficiently and equitably but differ in what is considered a resource (e.g., land, relationships, ceremony). Decision making is understood to be highly context-dependent in both paradigms. Western frameworks account for setting in evaluation design; Indigenous frameworks embed context as an expression of place, culture, and relational accountability. The concept of *context* aligns Western evaluation methods and engagement to Indigenous community leadership, knowledge, language, and Indigenous values. These concepts offer a foundational language for dialogue between Indigenous and Western approaches to health resource decision making.

The results of these thematic and sub-thematic linkages with their conceptual resonance between Western and Indigenous worldviews can be mapped into a novel framework (see Fig. 5). *Values* connect Western costs and resourcing, outcome measures, and engagement to Indigenous community strengths and leadership, knowledge, language, and medicines. *Holism* relates Western measurement, evaluation methods, equity, and intersectorality to Indigenous funding and policy impacts on community, medicines, and Indigenous values. *Time* links Western measurement, costs and resourcing, and investment with Indigenous community strengths, funding and policy impacts on community, Indigenous knowledge, and medicines. *Resources* connect Western costs and resourcing, evaluation methods, investment, equity, and intersectorality to Indigenous funding and policy impact on community, language, and Indigenous values. *Context* relates Western evaluation methods and engagement to Indigenous community leadership, knowledge, language, and Indigenous values.

## Discussion

Through a collaborative, community-engaged process with the I-HeLTI Health Economics Technical Advisory Group (HE-TAG), we identified conceptual and thematic areas of alignment between Indigenous and Western knowledge systems relevant to health resource decision making. These findings provide a foundation for advancing culturally grounded approaches to economic evaluation and outcome measurement in Indigenous health contexts. We employed a ‘two-eyed seeing’ approach to the concept and thematic analyses to understand the evidence that informs us of commonalities between Indigenous and Western health concepts in health resource decision making. This approach, coined by Mi’kmaq Elder Albert Marshall of the Eskasoni First Nation, involves seeing Indigenous ways of knowing through one eye and Western knowledge through the other, while alternating between vantage points [57]. A close reading of the central thematic content from each conceptual framework (summarized in Table 2) shows conceptual commonalities between Indigenous and Western frameworks. Applying two-eyed seeing [58–60] to the Indigenous and Western thematic content highlights shared themes and coherence in the perspectives they generate.

Our analysis revealed five core concepts from the thematic and concept analysis of ‘health’ from both a Western and Indigenous perspective, with their respective themes and subthemes. As “the very basis of any theory depends on the identification and explication of the concepts to be considered in it” [29], we see a new, conceptual theory of health resource decision making which includes the concepts of *values*, *holism*, *time*, *resources*, and *context* as common to both worldviews. While this specific grouping of concepts derives from the I-HeLTI HE-TAG discourse, evidence from the literature also supports the individual value of these concepts in health resource decision making.

Construction of new instruments to assess quality of life should strongly consider *values* with Indigenous applications [29]. Still, considering the example of Torres Strait Islander Indigenous peoples, measures have thus far failed to reflect their values [28], despite the frequency of using Western evaluative methods such as CCA, CEA, CUA, and CBA in these populations [61]. It seems the values that form the basis of various health economic evaluative measures are rarely described [12] since identifying the values at play that can empower participatory economics research is frequently challenging [62]. However, considering diversity of values is recognized to enhance the quality of social choices [63].

There have also been several efforts in health economics to adopt more holistic approaches to evaluate healthcare programs [22, 23, 28–30] and to acknowledge that *holism* is a characteristic of Indigenous culture concerning health and services [64]. For example, while holism is recognized in both systems, in Indigenous worldviews it is enacted through interdependence, where wellness is understood relationally—between individuals, families, land, and spirit [65]. In contrast, Western health economics often conceptualizes health domains as discrete variables that are measured independently. Similarly, both paradigms acknowledge the importance of *resources*, but Indigenous perspectives include land, language, and cultural knowledge as essential resources for health—dimensions often overlooked in conventional economic evaluation.

Recognizing these differences is critical not only for methodological adaptation but for ethical engagement. Applying Western models without contextual and cultural translation risks undermining Indigenous ways of knowing and reinforcing colonial assumptions about what counts as health and value. In the Canadian context, an evaluation of the First Nations Health Authority (FNHA) emphasized that holistic approaches toward Indigenous health also emphasize control over decision making at the community level [66], which highlights the connection between health sustainability and self-government.

Western conceptualizations of the linearity of *time* within the discipline of health economics are counterposed with other (i.e., spiral) Indigenous concepts [9]. Time trade-off (TTO) is a technique used in health economics to elicit health state utilities [67], and the UK utilizes TTO to derive values for the EQ-5D [68]. The stability and sensitivity of health-related quality of life (HRQoL) instruments to change over time have also been noted [5]. Time preference as it relates to discounting in models of social choice [69] is a key feature of health economic analysis. Moreover, opportunity cost depends on the time at which health benefits are shown [70]. These considerations generally tend to be of a shorter duration than the multigenerational timeframe of health impacts found in Indigenous worldviews.

The notion that health *resources* are scarce is commonly held in Western medical science [71], contrary to the pre-contact Indigenous understanding of resources. However, health care resource allocation can be guided by notions of equity and fairness [72]. From a Western point of view, resource decision making can either be based upon preferences, in which case QALYs can be calculated, or it can be based upon a valuation of subjective wellbeing [73]. Health resource allocation decisions can consider either treasury implications that have positive fiscal impacts, choose to increase social impacts and positive health outcomes, or balance fiscal and health outcomes in a blended approach [74]. In its most basic sense, resources are required to provide quality health care [75]. Although decision makers typically do not allocate health resources to other sectors [76], if a broader perspective is taken that considers inter-sectoral impacts, then resources within the wider economy could be considered as impacting health outcomes. FNHA recognizes the close linkage of health resources to the delivery of programs and services [66] while FNIGC indicates that the lack of adequate health resources and cultural appropriateness continue to be barriers to First Nations’ health [77].

The definition of costs and health effects depends on the *context* [78]. Equity considerations can be relevant for defining the decision problem [79]. Contextual relevance to decision making can have relative importance [80], and health technology assessment in general is seen as context-sensitive [81]. There is diversity in practical contexts where evidence is applied and can benefit from deliberative processes [72], and the context in which health programming is delivered can influence its effectiveness [75]. Incremental cost-effectiveness ratios (ICERs) will be context-dependent [82], and any chosen perspective taken in health economic analysis can influence the decision-making context [83]. Numerous economic evaluations of Australian Aboriginal and Torres Strait Islanders have taken health systems perspectives, partial societal perspectives, and societal perspectives [61]. The ever-present context of ongoing colonial impacts upon Indigenous populations with consequences to health outcomes based upon the SDH and the need to respond to the TRC’s Calls to Action in health [4, 77] cannot be overstated.

The resonance between Indigenous and Western knowledge systems through the five concepts (Fig. 5) occurs by understanding that when engaging with either one or the other conceptual subthematic group, the specific subtheme connects through individual, new themes to the opposing subthematic group. In this way, Indigenous and Western ways of health resource thinking resonate through the concepts of *values*, *holism*, *time*, *resources*, and *context*. This conceptual resonance should be seen as a positive indication that health economics and community decision making may find some manner of connecting, collaborating, and co-creating a meaningful Indigenous health resource evaluation metric, which may be used to support I-HeLTI programming and other Indigenous health programs. Practical examples of these results could include (1) using of the framework’s common themes to ground discussions with community on the co-development of health outcome measures; (2) demonstrating the potential value of health economics to community members; (3) modeling for health technology agencies the ways in which health measurement must be based on community values in order to be successful; and (4) applying core First Nations health values in priority-setting exercises in community.

In this analysis, we observed critical differences between Indigenous and Western concepts of ‘health’. The themes of *community* and *culture* produce and sustain health in the Indigenous context. The subthemes demonstrate an evident sustainability of health when considered together. Community strengths and leadership, knowledge, language, medicines, and Indigenous values are intrinsic to Indigenous health. Despite the challenges of funding and policy impacts upon communities which influence health, most subthemes are generative and sustaining; they are causative and broad contributors to health. By comparison, the Western themes that include *economic evaluation* and the *SDH* are primarily from the vantage point of seeing effects. Except for the investment of resources, which is generative, Western themes are primarily reactive. The act of measurement and using outcome measures, evaluation methods, equity, and intersectorality all take the vantage of assessing health after the fact. However, the *social determinants of health* theme also contains the ‘engagement’ subtheme. Engagement is seen as the forward path to connecting with participating I-HeLTI communities from understanding the SDH to connecting on the level of the sustaining elements of Indigenous health.

The ‘engagement’ subtheme can bring together holders of Indigenous and Western values to further advance conversations about how values, holism, time, resources, and context contribute to understanding health decision making. The following five principles for aligning values for health [84] can be used along this path to (1) create spaces for dialogue; (2) confront the paradoxes in how values are spoken about; (3) critically read between the lines of how values are framed; (4) zoom out to see the big picture on factors that shape health (i.e., SDH); and (5) move through many layers, which involves moving to the solution rather than being reactive.

Creating a space for dialogue involves engaging in values-based research with Indigenous communities by connecting with established Indigenous health values. Addressing the potential paradoxes within common themes that link Indigenous and Western health ideas can be achieved through ongoing community dialogue. For example, discussions about how both cultures can approach ‘time’ differently—such as linear versus cyclical views—highlight these differences. Additionally, using a community-based participatory research approach allows us to consider context, interpret underlying meanings, and understand specific cultural, linguistic, and health needs within communities. These discussions can then expand beyond just the health sector. Tackling the SDH requires recognizing the multisectoral influences on health outcomes, including education, housing, and infrastructure. The colonial impacts of funding and policy extend beyond the health sector alone and affect broader outcomes for Indigenous communities.

As demonstrated in the I-HeLTI HE-TAG discussions, moving to solutions will involve having community Elders lead university-based researchers in developing suitable methods. This practical application of the five resonating concepts between Indigenous and Western health can, therefore, be used as a path towards sustainable Indigenous health improvements based upon community values. It also shows how this novel conceptual theory may be applied in the future.

## Strengths and Limitations

This study demonstrates that it is possible—and necessary—to move toward ethical and culturally grounded health economics. The HE-TAG model illustrates a promising approach to supporting co-learning, conceptual alignment, and methodological innovation. One of the strengths of this novel theoretical framework is the potential to apply it to other Indigenous populations around the world. Despite being generated from a regionally specific area in Canada’s province of Alberta, its impact is expected to extend to international settings where the development of Indigenous health outcome measures is relevant. Indigenous leadership and participation during the I-HeLTI HE-TAG discussions also strengthen the data validity of the identified Indigenous health perspectives. Importantly, this work contributes to broader efforts to reorient health systems and policies toward equity, reconciliation, and Indigenous self-determination. Limitations of this research include that the proposed theoretical framework has not yet been tested and validated beyond the Maskwacîs community, where it was presented and discussed with their community advisory committee in March 2025. Initial indications of the framework’s meaningfulness during this engagement with a First Nation were positive. Also, the discursive data from this case study may not have identified the full range of resonating concepts between Indigenous and Western health resource decision making. Further direct community engagement should be undertaken to mitigate these limitations and build upon the strengths of this work.

## Conclusion

This study explored areas of resonance between Indigenous and Western frameworks of health and resource decision making to inform the development of more culturally relevant and ethically grounded approaches to health outcome measurement and economic evaluation. Through a collaborative, multi-method analysis of dialogue from the I-HeLTI Health Economics Technical Advisory Group, we identified five shared concepts—values, holism, time, resources, and context—that offer a bridge between knowledge systems while also highlighting important distinctions in meaning and application.

These findings underscore the urgent need to rethink how health is defined, measured, and valued within policy and funding decisions affecting Indigenous Peoples. Relying on conventional health economics tools without adaptation risks reinforcing structural inequities and cultural misalignment. Instead, integrating Indigenous knowledge systems into the design of evaluation frameworks, outcome measures, and decision-making processes is essential for supporting health system transformation that is responsive, relational, and rooted in Indigenous self-determination. In this way, sustainable improvements in Indigenous health outcomes through programming such as the I-HeLTI can be measured in understandable, holistic ways for the benefit of community decision makers. This work is also relevant to governmental policy decision makers who wish to approach economic evaluation of Indigenous health programming in a culturally sensitive manner.

## Declarations

### Data Availability Statement

The HE-TAG data that supports this study are available from the Collaborative Applied Research for Equity in Health Policy and Systems (CARE) Lab, but restrictions apply to the availability of these data. The data are, however, available from the authors upon reasonable request and with the permission of the CARE Lab.

### Authors’ expertise

The strength of this work is grounded in the diverse and complementary expertise of the eight co-authors, whose leadership spans Indigenous health systems, health economics, community-based research, and policy development. Stephanie Montesanti is a nationally recognized expert in Indigenous health systems research, with specialized expertise in Indigenous research methodologies, systems thinking, health system integration, and intersectoral policy analysis. She is a Professor and Canada Research Chair in Health Systems Integration, leading the Collaborative Applied Research for Equity in Health Policy and Systems (CARE) Lab at the University of Alberta. As a Principal Investigator of the I-HeLTI Alberta team, Dr Montesanti provides strategic vision for advancing equity-centered and values-based approaches to health system transformation. Lea Bill is an Indigenous knowledge holder from the Cree Pelican Lake First Nation in Saskatchewan with thirty years of experience in community as a registered nurse. Lea is also Executive Director of the Alberta First Nations Information Governance Centre (AFNIGC) where she is also a project lead and research manager. Barbara S.E. Verstraeten holds a medical degree and a PhD in health sciences, with a focus on pregnancy, maternal, and child health, as well as the developmental origins of health and wellness. Barbara is a research associate in the CARE lab, supporting qualitative and quantitative data analysis for the I-HeLTI and other projects. Rhonda Bell is a professor of human nutrition in the Faculty of Agricultural, Life & Environmental Sciences at the University of Alberta. Rhonda is an expert in maternal and infant nutrition and health outcomes with a focus on the developmental origins of health and disease. Richard Oster is Scientific Director, Indigenous Wellness Core with the government of Alberta, and he has worked with and learned from Indigenous communities, particularly relating to engagement, collaboration, community-based participatory research, epidemiology, qualitative inquiry, intervention design, project management, knowledge translation, Indigenous ways of knowing, and Indigenous research ethics. Arto Ohinmaa is a professor of health economics at the University of Alberta with expertise in economic evaluation, health-related quality of life, health outcomes, cost-effectiveness analysis, and health services research. Mike Paulden has expertise in cost-effectiveness analysis and developing appropriate methods for the economic evaluation of health technologies. Mike is also interested in how social values can be integrated within the methods used for health economic evaluations. Aidan Neill is a recent PhD graduate in health services and policy research. Aidan is a resource professional and chartered professional accountant (CPA) who has worked in the Canadian federal policy space for over twenty years.
